# Increased IDO expression and regulatory T cells in acute myeloid leukemia: implications for immune escape and therapeutic targeting

**DOI:** 10.1007/s44313-024-00048-0

**Published:** 2024-12-18

**Authors:** Raziyeh Hakak, Behzad Poopak, Ahmad Majd

**Affiliations:** 1 Department of Cellular and Molecular Biology, Faculty of Biological Sciences, North Tehran Branch, Islamic Azad University, Tehran, Iran; 2 Payvand Clinical and Specialty Laboratory, Tehran, Iran

**Keywords:** Acute myeloid leukemia, Flow cytometry, Indoleamine 2,3-dioxygenase, Real-time polymerase chain reaction, T regulatory cells

## Abstract

**Purpose:**

This study aimed to determine the frequency of regulatory T cells (Tregs) (CD4^+^/FOXP3^+^) and indoleamine 2,3-dioxygenase (IDO) expression in patients with acute myeloid leukemia (AML).

**Methods:**

This cross-sectional case–control study was conducted between Jan 2022 and Dec 2023. Bone marrow samples were collected from 20 healthy individuals and 15 patients with AML. Flow cytometry, real-time polymerase chain reaction (PCR), and western blotting were used to evaluate the frequency of Treg and IDO expression levels.

**Results:**

The Treg percentage among total lymphocytes was lower in the AML group than that in the normal group. However, Treg percentage among T-helper (Th) lymphocytes was significantly higher in the AML group than that in the normal group (*p* < 0.05). The mean IDO expression in the AML group was significantly higher than that in the normal group (*p* = 0.004). A significant relationship was observed between IDO expression and Treg percentage among Th lymphocytes in the AML group (correlation = 0.637; *p* = 0.003). Moreover, western blot analysis showed a significant increase in IDO protein intensity in the AML group compared with that in the control group (*p* < 0.001). A significant difference was observed between the IDO concentrations in the AML group and that in the control group (*p* < 0.001). In addition, a significant difference between TGF-β levels in the AML group and those in the control group (*p* < 0.01) was observed.

**Conclusion:**

IDO inhibition using novel IDO inhibitors along with chemotherapy is a promising approach to overcome the immune escape mechanisms in patients with AML, who exhibit increased levels of IDO expression and Tregs.

## Introduction

Acute myeloid leukemia (AML) affects the bone marrow (BM). Under physiological conditions, the BM facilitates the production and trafficking of immune cells, especially those of the myeloid lineage [1]. AML is the most prevalent myeloid leukemia in adults and is the most common form of acute leukemia [2]. The various types and subtypes of these malignancies may arise from distinct pathogenic mechanisms, suggesting a functional correlation between different genetic and molecular abnormalities, as well as gene expression patterns, and disease prognosis [3, 4]. Studies on immune responses in patients with AML generally show an increase in immune inhibitory activity, which is one of the primary factors in pathogenesis and disease progression [5, 6].

Among immune cells, the number and function of regulatory T lymphocytes (Tregs) are associated with recurrence and poor prognosis in these patients; therefore, the inhibition or reduction of Tregs may enhance the efficiency of treatment and immunotherapy [7, 8].

Tregs are the primary cells that regulate the immune system and play an important role in maintaining self-tolerance. Comprehending the biology of these cells is valuable for their precise recognition and their manipulation serves as a pivotal component in the exploration of novel immunotherapy approaches [9]. FoxP3 has been identified as a major regulator of Treg evolution and function, and mutations or inactivation of FoxP3 can cause immune disorders [10]. FoxP3 activates genes such as CD25, CTLA-4, and GITR in Tregs, leading to their suppressive activity [11].

Indoleamine 2,3-dioxygenase (IDO) is a novel immunosuppressive agent expressed in certain subtypes of normal and neoplastic cells, including AML cells. IDO expression is associated with an increase in Tregs (CD4^+^CD25^+^FOXP3^+^). In vitro, IDO^+^ AML cells increase the number of CD4^+^CD25^+^ T cells expressing FoxP3 mRNA. IDO is a key enzyme in tryptophan metabolism, catalyzing the initial and rate-limiting step of tryptophan degradation through the kynurenine pathway [12]. Tryptophan deficiency inhibits T cell activation with IDO intake, whereas metabolites of tryptophan catabolism, such as kynurenine derivatives and oxygen-free radicals, regulate cell proliferation and survival. Therefore, IDO exerts immunosuppressive effects within the tumor microenvironment [13].

Several studies have shown a direct and reciprocal relationship between the production and activity of IDO and the number and function of Tregs. Furthermore, elevated IDO levels have been observed in some cancer patients, contributing to the inhibition of immune responses [14]. The present study aimed to evaluate the frequency of CD4^+^CD25^+^FoxP3^+^ Tregs and IDO expression in patients with AML.

## Methods

### Sample collection and preparation

This cross-sectional case–control study was conducted between January 2022 and December 2023. BM samples were collected from 15 patients diagnosed with AML and 20 healthy individuals who served as controls. All participants were referred to the hematology department of Payvand Clinical & Specialty Laboratory. For all participants, demographic details, including age and sex, were recorded, along with clinical parameters such as AML subtype and blast percentage for the patient group. All samples were processed within 2 h of collection to ensure optimal cell viability.

### Flow cytometry for Treg assessment

To assess the percentage of Tregs in BM samples, flow cytometry was performed using specific antibodies. Small aliquots (100 μL) of whole blood containing K3EDTA were incubated with anti-CD3 (FITC), CD4 (Krome Orange), CD8 (Pacific Blue), CD25 (PE), CD45 (PerCP), CD127 (PE-Cy7), and FoxP3 (Alexa 647) antibodies (BD Pharmingen, USA) for 30 min at room temperature, protected from light. Red blood cells were lysed using a lysing solution (BD Biosciences, USA), and the remaining cells were fixed and permeabilized using the Human FoxP3 Buffer Set (BD, USA). After surface and intracellular staining, the cells were analyzed using a BD FACSCanto II flow cytometer (BD Biosciences). Gating was performed on CD3^+^CD4^+^ cells, with Tregs identified as CD25^high^CD45^+^CD127^low^FoxP3^+^ cells. Isotype controls were used to determine background staining.

### RNA extraction and cDNA synthesis

Total RNA was extracted from isolated Tregs using the RNX Plus kit (Cinnagen, Iran) according to the manufacturer’s instructions. Approximately 5 × 10^5^ cells were used for RNA extraction. RNA concentration and purity were determined using a Nanodrop spectrophotometer, with samples showing an A260/A280 ratio of 1.9–2 considered pure. RNA integrity was confirmed using electrophoresis, where sharp 28S and 18S rRNA bands with a 2:1 intensity ratio indicated intact RNA. For cDNA synthesis, 500 ng of total RNA from each sample was treated with RNase-free DNase (Qiagen, USA) to remove genomic DNA contamination. First-strand cDNA was synthesized using the RevertAid™ First Strand cDNA Synthesis Kit (Thermo Fisher Scientific, USA) with random hexamer primers. Successful cDNA synthesis was confirmed using polymerase chain reaction (PCR) amplification of glyceraldehyde-3-phosphate dehydrogenase (GAPDH) transcripts.

### Real-Time Quantitative Reverse Transcription-Polymerase Chain Reaction (qRT-PCR)

Quantitative real-time PCR (qRT-PCR) was performed to assess the expression of IDO, a key experimental factor, and the housekeeping gene albumin (Alb). Primers for these genes were designed using the Gene Runner software and synthesized by Tag Copenhagen (Denmark) (Table 1). qRT-PCR was conducted using the SYBR Green Master Mix (Thermo Fisher Scientific, USA) on a Rotor-Gene Q real-time PCR system (Qiagen, Germany). The PCR program consisted of an initial denaturation at 95 °C for 10 min, followed by 40 cycles of 95 °C for 30 s, 59 °C for 30 s, and 72 °C for 35 s. A melting curve analysis was performed from 72 °C to 95 °C to verify the specificity of the PCR products. PCR products were analyzed by electrophoresis on a 2% agarose gel and visualized under ultraviolet (UV) light.

**Table 1:** Primers and reaction conditions used in qRT-PCR

Gene	Sequence	Product size (bp)
IDO	F: 5′-TCCTGGACAATCAGTAAAGAGTACC-3′ R: 5′-TCAGGCAGATGTTTAGCAATGAAC-3′	122
Albumin	F: 5′-GCTATCCGTGGTCCTGAACC-3′ R: 5′-CTTCTCAGAAAGTGTGCATATATCTG-3′	200

### Isolation of Tregs and cytokine analysis

Mononuclear cells were separated from the BM samples by density gradient centrifugation using Ficoll-Paque PLUS (GE Healthcare, USA). Thereafter, the cells were isolated using magnetic-activated cell sorting. The cells were then labeled with anti-CD4, anti-CD25, and anti-CD127 magnetic beads (Miltenyi Biotec, Germany), according to the manufacturer’s instructions. The labeled cells were passed through a magnetic column to separate Tregs from other cell types. The purity of the isolated Tregs was confirmed to be greater than 95% using flow cytometry. Subsequently, CD4^+^CD254^high^CD127^low^FoxP3-positive and CD4^+^CD254^high^CD127^low^-negative T cells were purified using a FACSCaliber cell sorter (Becton Dickinson). To measure TGF-β and IDO, Tregs were stimulated with phorbol myristate acetate (PMA) (55 ng/mL) in the presence of GolgiPlug (BD Biosciences) for 5 h at 37 °C before enzyme-linked immunosorbent assay (ELISA).

All ELISA tests were conducted to measure the concentrations TGF-β in Treg culture supernatants. ELISA kits for TGF-β (R&D Systems, USA) were used according to the manufacturer’s instructions. Samples were added to pre-coated plates and incubated for 2 h at room temperature. After washing, detection antibodies were added and incubated for another 2-h incubation. The substrate solution was then added and the reaction was stopped with a stop solution. Absorbance was measured at 450 nm using a microplate reader (BioTek, Winooski, VT, USA), and cytokine concentrations were calculated based on standard curves of recombinant cytokines.

### Protein expression analysis using western blotting

To complement the gene expression data, protein expression levels of IDO and transforming growth factor beta (TGF-β) in Tregs were analyzed using western blotting. Total protein was extracted from isolated Tregs using radioimmunoprecipitation assay (RIPA) buffer (Thermo Fisher Scientific) containing a protease inhibitor cocktail (Roche, Switzerland). Protein concentration was measured using a bicinchoninic acid assay kit (Thermo Fisher Scientific, USA). Equal amounts of protein (30 μg per sample) were separated using sodium dodecyl sulfate–polyacrylamide gel electrophoresis on a 10% polyacrylamide gel and transferred onto a polyvinylidene fluoride (PVDF) membrane (Millipore, USA). Membranes were blocked with 5% non-fat dry milk in Tris-buffered saline with Tween 20 for 1 h at room temperature, then incubated overnight at 4 °C with primary antibodies against IDO (1:1000, Abcam, USA) and GAPDH (1:5000, Cell Signaling Technology, USA). After washing, the membranes were incubated with horseradish peroxidase -conjugated secondary antibodies (1:2000; Cell Signaling Technology) for 1 h at room temperature. The bands were visualized using an enhanced chemiluminescence detection system ( GE Healthcare, USA) and quantified using the ImageJ software.

### Statistical analysis

Statistical analyses were performed using Statistical Package for the Social Sciences (SPSS) version 26 (SPSS Inc., Chicago, Illinois, USA). Data are expressed as mean ± standard deviation for continuous variables and as frequencies (percentages) for categorical variables. The normality of the data distribution was assessed using the Kolmogorov–Smirnov test. Differences between groups were analyzed using the independent t-test or Mann–Whitney U test, as appropriate. Correlations were evaluated using the Spearman’s rank correlation coefficient. Statistical significance was set at *p* < 0.05.

### Ethics statement

This study was reviewed and approved by the Ethics Committee of Azad University (approval no. IR.IAU.TNB.REC.1401.008). This study was conducted in accordance with the principles of the Declaration of Helsinki.

## Results

**Fig. 1:**
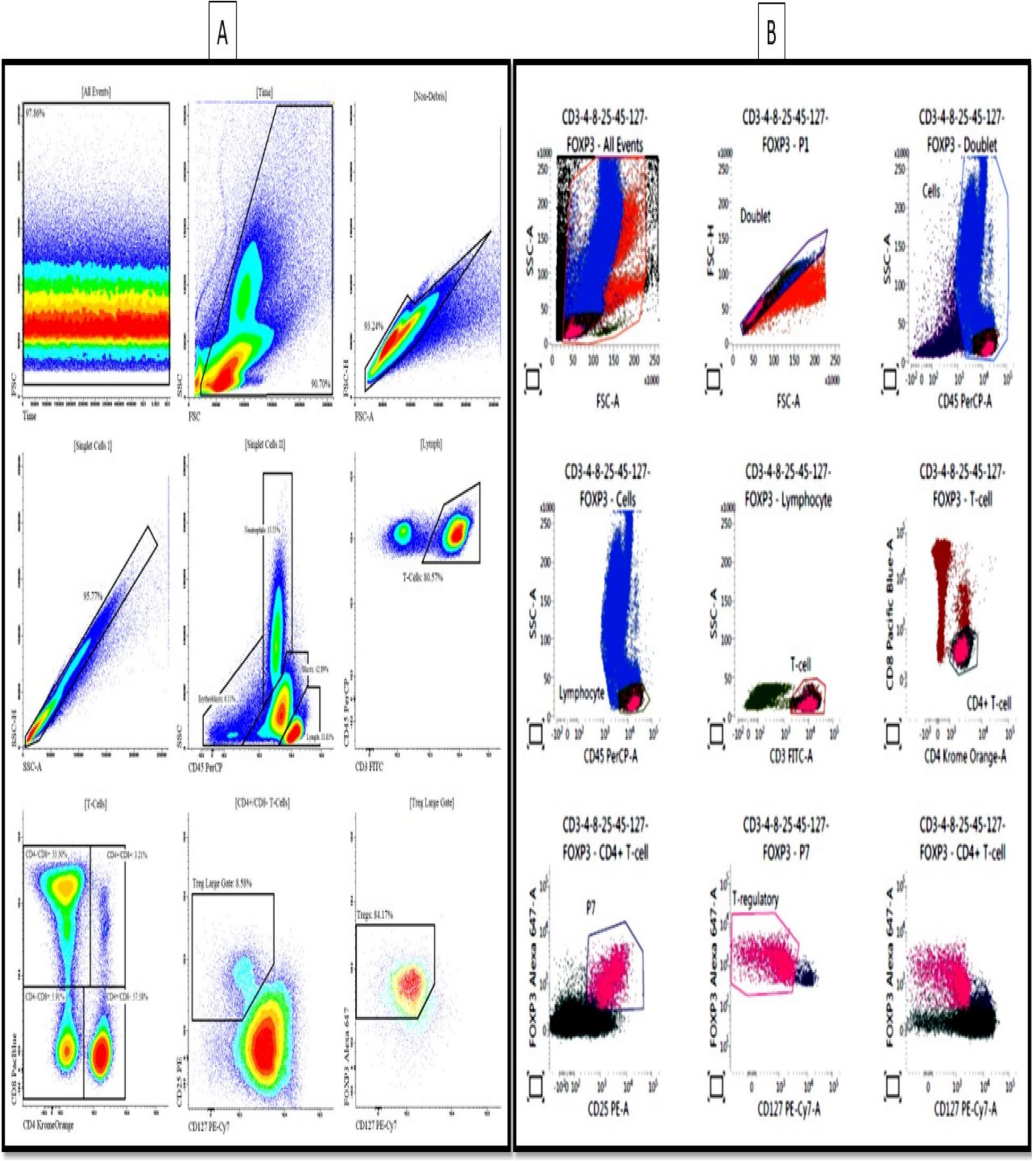
Gating strategy (**A**) and frequency of CD4^+^CD25^+^FoxP3.^+^ Tregs (**B**)

Of the 20 patients in the normal group, 13 (65%) were male and seven (35%) were female. Among the 15 patients with AML, four (26.7%) were male, and 11 (73.3%) were female. The demographic characteristics of the study participants are presented in Table 2. The most frequent cell type observed in Non-M3 AML was common B cells (33.3%, *p* < 0.001). Flow cytometry analysis revealed that the CD25^high^CD45^+^CD127^low^FoxP3^+^ Treg percentage in total lymphocytes was lower in the AML group (0.55 ± 0.56) than that in the normal group (1.72 ± 0.88). However, the Treg percentage in Th lymphocytes was significantly higher in the AML group (9.01 ± 2.53) than that in the normal group (7.25 ± 2.91) (*p* < 0.05; Fig. 1).

**Table 2:** Basic and clinical characteristics of patients in the two groups

Characteristics	Normal group	AML group	*p*-value
Gender			
**Male**	13 (65%)	4 (7.26%)	_0.023_
**Female**	7 (35%)	11 (3.73%)	
Age, year	33.27 ± 90.28	33.24 ± 00.37	≥ 0.001
Less than 5 years (toddler)	4 (20%)	2 (3.13%)	
5–13 years (child)	6 (30%)	2 (3.13%)	
14–19 years (adolescent)	1 (5%)	0 (0%)	≥ 0.001
20–55 years (adult)	3 (15%)	7 (7.46%)	
Over 55 years (older adult)	6 (30%)	4 (7.26%)	
Subtype			
Common B	0 (0%)	0 (0%)	
M0	0 (0%)	1 (7.6%)	
M1	0 (0%)	4 (7.26%)	
M1/M2	0 (0%)	1 (7.6%)	
M2	0 (0%)	1 (7.6%)	
M4/M2	0 (0%)	1 (7.6%)	≥ 0.001
M5	0 (0%)	2 (3.13%)	
Non-M3	0 (0%)	5 (3.33%)	
Pro B	0 (0%)	0 (0%)	
Relapsed B	0 (0%)	0 (0%)	
Relapsed T	0 (0%)	0 (0%)	
T	0 (0%)	0 (0%)	
Blast percentage	53.0 ± 87.0	99.28 ± 13.5	≥ 0.001

**Fig. 2:**
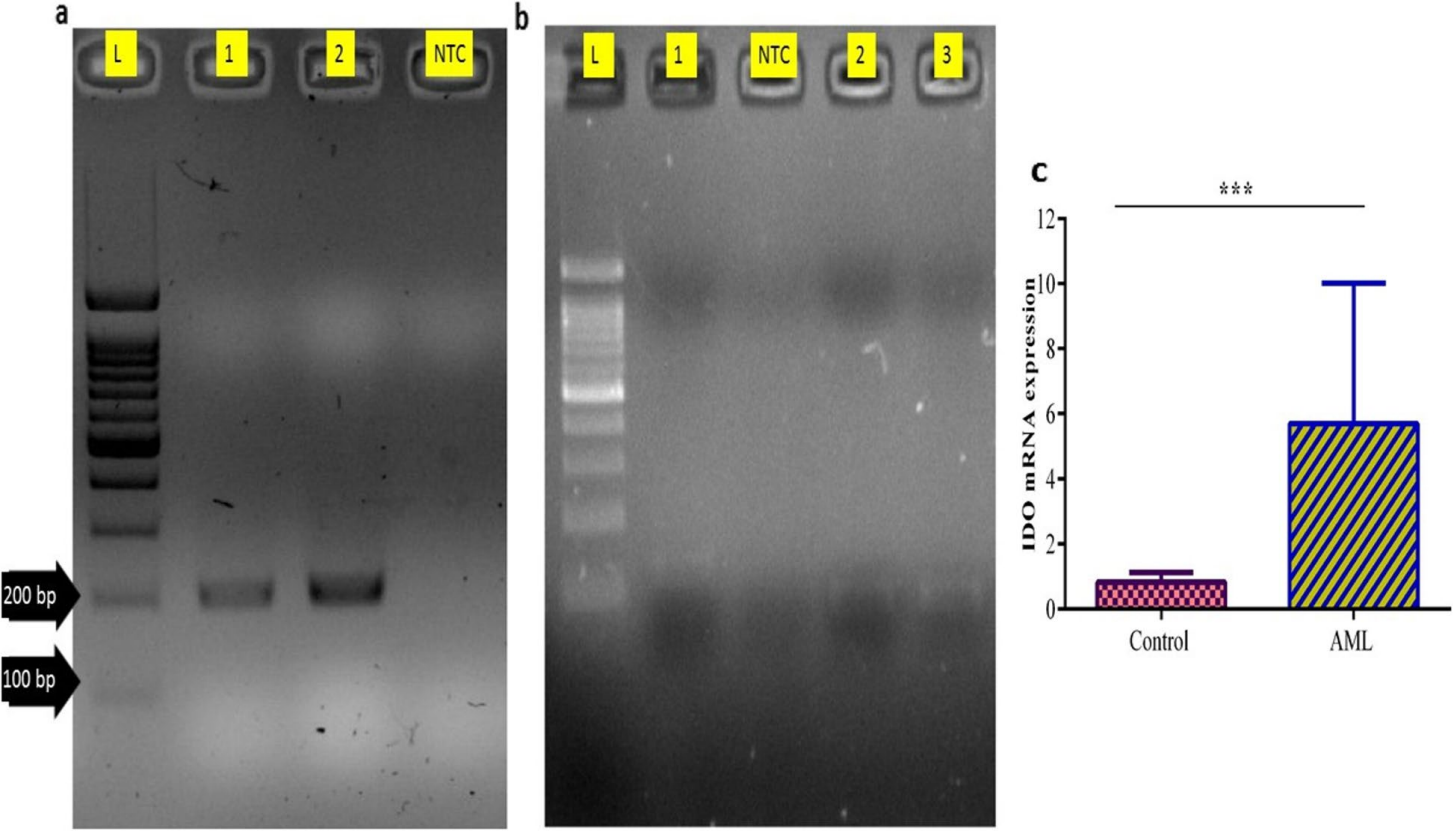
**a** The Albumin qRT-PCR products analyzed using a 2% agarose gel. Results confirmed the expected unique 200 bp band for the Albumin gene, indicating specific amplification of Albumin. L: 100 bp ladder (Fermentas), 1& 2: analytical samples, NTC: no template control. **b** The IDO qRT-PCR products analyzed using a 2% agarose gel. The results confirmed the expected unique 122 bp band for the IDO gene, indicating specific IDO amplification. L: 100 bp ladder (Fermentas); 1, 2 & 3: analytical samples, NTC: no template control. **c** Comparison of mRNA levels of IDO between AML and control groups. (***p < 0.001)

**Fig. 3:**
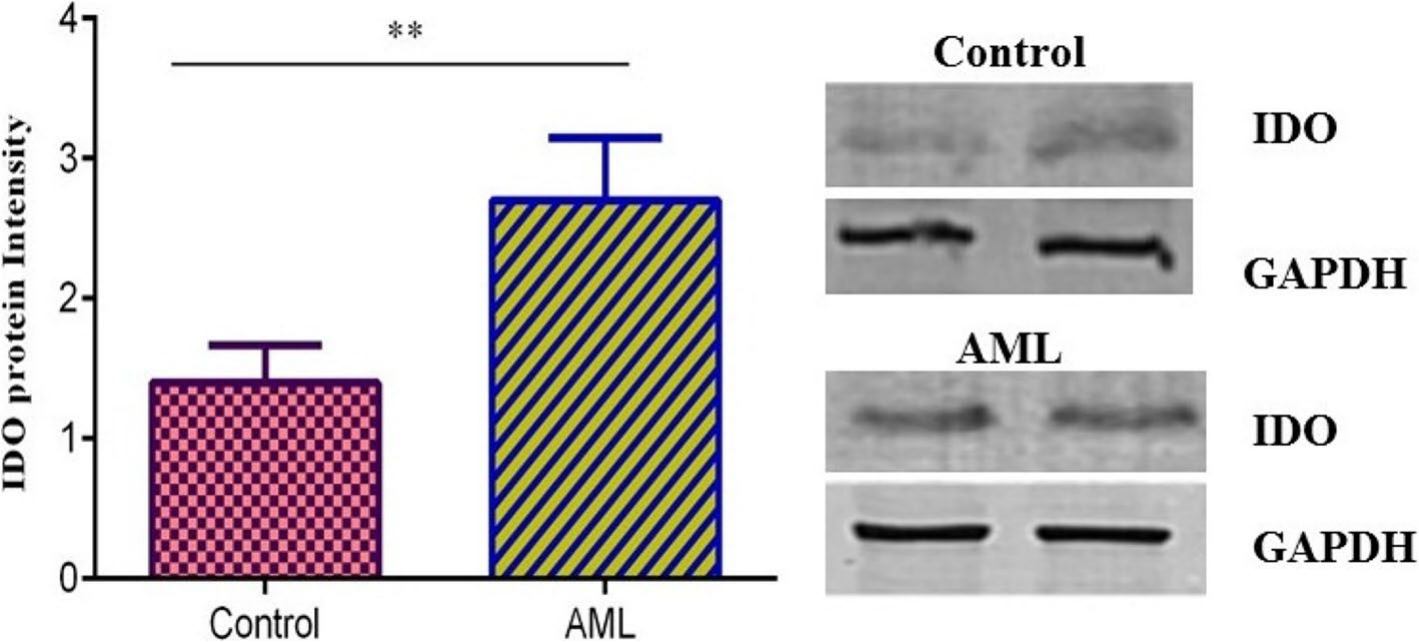
Analysis of IDO protein intensity in the AML and control groups using western blotting (**p < 0.01)

The qRT-PCR melting curve results showed specific amplification of the IDO and Alb genes. Electrophoresis on a 2% agarose gel confirmed unique bands corresponding to IDO and Alb genes (Fig. 2a-b). The qRT-PCR analysis showed that mean expression of IDO was significantly lower in the normal group (0.92 ± 1.32) than that in the AML group (4.69 ± 6.01) (*p* = 0.004) (Table 3, Fig. 2c). Moreover, western blot analysis showed a significant increase in IDO protein intensity in the AML group compared with that in the control group (*p* < 0.001; Fig. 3). Analysis of the relationship between IDO expression and Treg percentages revealed a significant correlation between IDO expression and the Treg percentage in Th lymphocytes in the AML group, with a correlation coefficient of 0.637 (*p* = 0.003). In contrast, although a direct relationship between IDO expression and different percentages of Treg was observed in the normal group, this relationship was not statistically significant (*p* > 0.05) (Table 4).

**Table 3:** Comparison of mean percentages of Tregs in patients in the two groups

Treg percentage	Normal group	AML group	*p*-value
**Percentage of total Tregs**	00.0 ± 69.0	0.00 ± 55.0	0.309
**Percentage of Tregs in total lymphocytes**	1.00 ±72.1	00.0 ± 55.56	0.023
**Percentage of Tregs in T lymphocytes**	3.1 ± 32.99	4.1 ± 28.3	0.134
**Percentage of Tregs in Th lymphocytes**	7.2 ± 25.97	9.2 ± 01.53	0.030

**Table 4:** Correlation coefficients of IDO expressions with the percentages of Tregs

Percentage Treg	Normal group		AML group	
	Correlation coefficient	*p*-value	Correlation coefficient	***p*-value**
**Percentage of total Tregs**	083.0	0.729	246.0	0.377
**Percentage of Tregs in total lymphocytes**	350.0	0.130	305.0	0.269
**Percentage of Tregs in T lymphocytes**	251.0	0.286	191.0	0.494
**Percentage of Tregs in Th lymphocytes**	637.0	0.003	164.0	0.559

**Fig. 4:**
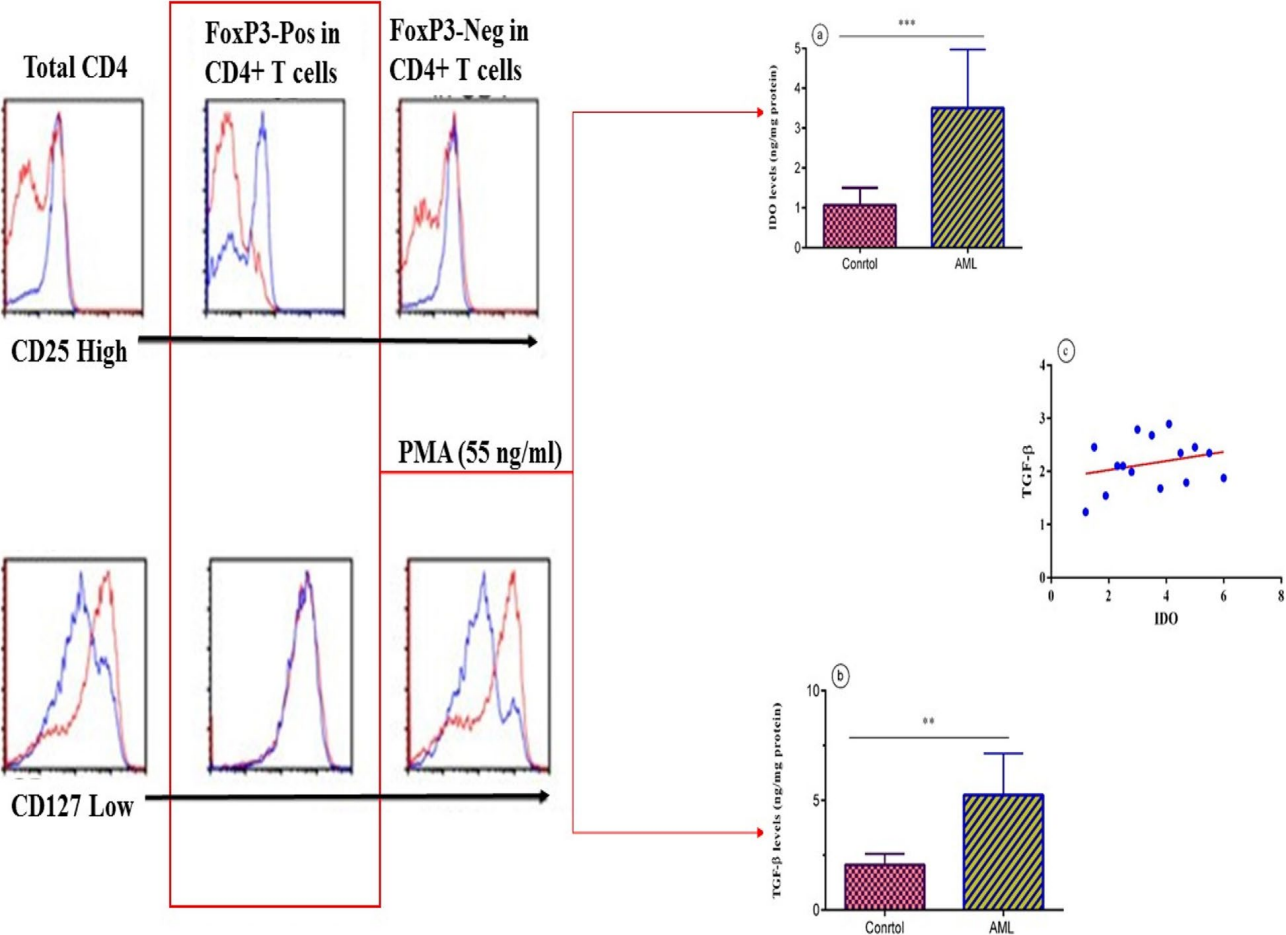
Concentrations of IDO (**a**) and TGF-β (**b**) in the bone marrow of the AML and control groups. No significant correlation is observed between IDO and TGF-β levels (**c**) (**p < 0.01, ***p < 0.001)

There was a significant difference in concentrations of IDO between the AML group (3.136 ± 1.845 ng/mL) and the control group (1.076 ± 0.369 ng/mL) (*p* < 0.001; Fig. 4a). Similarly, a significant difference was observed in TGF-β levels between the AML group (5.098 ± 1.522 ng/mL) and the control group (2.306 ± 0.471 ng/mL) (*p* < 0.01, Fig. 4b). However, no significant correlation was seen between IDO and TGF-β levels in AML group (*p* < 0.345, r = 0.069; Fig. 4c).

## Discussion

In this study, we investigated the relationship between Tregs and IDO expression in patients with AML. Our findings showed that the percentage of Tregs among Th lymphocytes was significantly higher in patients with AML than that in healthy controls, whereas the total Treg percentage was lower in patients with AML compared with that in control group. Additionally, IDO expression and protein levels were significantly elevated in the AML group. A strong correlation between IDO expression and Treg percentages was found in AML group, suggesting a potential immune evasion mechanism. The present study also revealed higher levels of IDO and TGF-β in the AML group compared with those in control group, although no significant correlation between IDO and TGF-β was observed. These results provide new insights into the immunosuppressive environment in AML and highlight the potential of IDO and Tregs as therapeutic targets.

Overall, the percentage of men was higher in the normal group than in the AML group. In addition, the mean age of participants was much higher in the AML group than that in the normal group. Similarly, based on previous epidemiological reports, AML is the most common myeloid leukemia in adults and the most common type of acute leukemia [15]. The prevalence of AML increases with age [16, 17], and this condition is more commonly observed in boys than in girls; this difference is more pronounced in older patients [18].

In the present study, although the AML group had a higher mean age, a higher percentage of women were affected compared with that in the normal group. This sex disparity may be due to the small sample size, as larger epidemiological studies generally report a higher incidence of AML in males than in females [19]. Further research with a larger cohort is needed to clarify this gender-related observation.

Examining the Treg cell percentage revealed that while the percentages of total Tregs and T lymphocytes were not significantly different between the normal and AML groups, the percentage of Tregs in total lymphocytes was significantly lower in AML group, whereas the percentage in Th lymphocytes was significantly higher compared with that in the normal group. Mansour et al. also showed a significant increase in the percentage of Tregs in patients with AML compared with that in the control group [20]. In addition, Moon et al. found that the population of Tregs in the peripheral blood and BM increased significantly in AML and high-grade MDS groups compared with those in controls [21], which can be explained by previous findings suggesting that AML cells secrete factors that inhibit the activation and proliferation of T cells and limit the production of anti-inflammatory cytokines such as those from Th1 cells [22, 23]. The higher proportion of Tregs among Th lymphocytes in patients with AML may contribute to immune evasion, suggesting that therapeutic strategies aimed at reducing Treg levels in this subpopulation could enhance antitumor immunity. These findings are consistent with previous reports indicating that targeting Tregs in patients with AML can improve the efficacy of immunotherapies [24, 25].

When TBs and other T lymphocytes are released from the microenvironment, this suppressive effect is reversed, leading to an enhanced immune response in AML [26]. This can be explained by the effect of mesenchymal stem cells (MSC) in the brain microenvironment on the production of CD4^+^CD25^+^FoxP3^+^ cells. Ivanova-Todorova et al. observed an increase in the stability of CD4^+^CD25^+^FoxP3^+^ cells in the presence of MSCs compared with the corresponding control media without MSCs [27].

De Matteis et al. also showed that the proportion of Tregs increased in all patients with chronic lymphocytic leukemia, along with a significant increase in IL-10 and IL-4 levels [28]. Evaluation of IDO expression showed that IDO expression was significantly higher in the AML group than that in the normal group. Healthy individuals exhibited the lowest IDO expression. Similarly, Arandi et al. reported the highest IDO expression in patients with AML compared with that in the normal group, suggesting that high IDO expression, along with increased FoxP3 expression levels (indicating an increase in the Treg phenotype), contributed to the poor immune response of patients with AML, disease progression to later stages, and poor prognosis [12]. The elevated IDO protein levels in patients with AML observed in our study, confirmed by western blot analysis, further support the notion that IDO contributes significantly to immune suppression in AML. Western blotting provides a more direct measure of protein expression, highlighting the role of IDO as a potential target for therapeutic intervention [14].

Mansour et al. also showed a significant increase in IDO expression in the MSCs of patients with AML compared with that in the control group. This increased expression can be attributed to the ability of leukemic cells to induce changes in MSCs [20]. Civini et al. demonstrated a dynamic relationship between BM-MSCs and leukemic cells. They confirmed that BM-MSCs affect leukemia cells and found that leukemic cells alter the profile of cytokines produced by BM-MSCs through proinflammatory agents, without requiring direct contact between BM-MSCs and leukemic cells [29]. Therefore, IDO inhibition, with a focus on the use of new IDO inhibitors along with chemotherapy, can be an acceptable solution to overcome immune escape mechanisms in patients with AML, who experience increased levels of IDO expression. Recent studies have suggested that dual inhibition of IDO and other immunosuppressive pathways may have synergistic effects, enhancing the overall antitumor immune response in AML [14]. Interestingly, our study found no significant correlation between IDO and TGF-β levels in AML patients, although both were elevated compared to those in controls. This lack of correlation suggests that IDO and TGF-β may operate through independent mechanisms to mediate immune suppression in the AML microenvironment. TGF-β is known to induce Tregs and suppress effector T cell function [30], but its exact relationship with IDO expression in AML requires further investigation. Future studies should explore the crosstalk between these two pathways to develop more comprehensive therapeutic strategies targeting immune suppression in AML.

Curti et al. reported an increase in Treg levels in leukemic patients whose AML cells expressed IDO. They found that AML cells expressing IDO (possibly by inducing FoxP3 expression) could convert normal CD4^+^CD25^−^ T cells into CD4^+^CD25^+^ Tregs, which was completely abolished in vitro by inhibiting IDO via 1-methyl-tryptophan (a competitive inhibitor of IDO) [31]. Consistent with this, Curti et al. observed that IDO-expressing dendritic cells disrupted the specific immune response to leukemia by enhancing the development of regulatory T cells [32]. Direct inhibition of T cell proliferation and the induction of the Treg phenotype are responsible for the immune escape mechanism used by patients with AML to produce IDO [33]. The positive correlation observed in this study between IDO expression and Treg percentage in Th lymphocytes in patients with AML underscores the role of IDO in Treg induction, reinforcing the idea that IDO inhibitors can potentially disrupt this immunosuppressive axis [32]. This finding supports ongoing research into IDO inhibitors, such as epacadostat, as adjuvant therapies to standard AML treatments [34].

However, due to incomplete data for some patients and limited access to their clinical data, it was not possible to pursue their clinical outcomes in terms of disease progression or survival, or to determine the prognostic significance of changes in IDO/Foxp3 expression. Studying a larger population of patients with accessible and complete data can help overcome this limitation and may advance our understanding of the importance of IDO prognosis and managing clinical outcomes of patients.

## Conclusion

Based on the results of our study, the frequency of CD4^+^CD25^+^FoxP3^+^ Tregs and IDO expression were significantly higher in patients with AML compared with those in the normal group. These findings may be responsible for poor immune response in these patients, disease progression to later stages, and poor prognosis. Therefore, IDO inhibition, using novel IDO inhibitors along with chemotherapy, may be a promising approach to overcome the immune escape mechanisms in patients with AML, who exhibit increased levels of IDO expression and Tregs.

## Data Availability

No datasets were generated or analysed during the current study.
